# Inhibition of castration-resistant prostate cancer growth by genistein through suppression of AKR1C3

**DOI:** 10.29219/fnr.v67.9024

**Published:** 2023-01-31

**Authors:** Xiaoping Yu, Jiali Yan, Yulu Li, Jing Cheng, Lujie Zheng, Tianyu Fu, Yanfeng Zhu

**Affiliations:** 1 School of Medicine and Nursing, Chengdu University, Chengdu, China; 2 School of Public Health, Chengdu Medical College, Chengdu, China; 3 National Institute for Occupational Health and Poison Control, Chinese Center for Disease Control and Prevention, Beijing, China

**Keywords:** genistein, castration-resistant prostate cancer, AKR1C3, prostate cancer, Phytochemicals

## Abstract

**Background:**

Prostate cancer is the second leading cause of cancer-related death among males in America. The patients’ survival time is significantly reduced after prostate cancer develops into castration-resistant prostate cancer (CRPC). It has been reported that AKR1C3 is involved in this progression, and that its abnormal expression is directly correlated with the degree of CRPC malignancy. Genistein is one of the active components of soy isoflavones, and many studies have suggested that it has a better inhibitory effect on CRPC.

**Objective:**

This study aimed to investigate the antitumor effect of genistein on CRPC and the potential mechanism of action.

**Design:**

A xenograft tumor mouse model established with 22RV1 cells was divided into the experimental group and the control group, and the former was given 100 mg/kg.bw/day of genistein, with 22RV1, VCaP, and RWPE-1 cells cultured in a hormone-free serum environment and treated with different concentrations of genistein (0, 12.5, 25, 50, and 100 μmol/L) for 48 h. Molecular docking was used to elucidate the molecular interactions between genistein and AKR1C3.

**Results:**

Genistein inhibits CRPC cell proliferation and in vivo tumorigenesis. The western blot analysis confirmed that the genistein significantly inhibited prostate-specific antigen production in a dose-dependent manner. In further results, AKR1C3 expression was decreased in both the xenograft tumor tissues and the CRPC cell lines following genistein gavage feeding compared to the control group, with the reduction becoming more obvious as the concentration of genistein was increased. When the genistein was combined with AKR1C3 small interfering ribonucleic acid and an AKR1C3 inhibitor (ASP-9521), the inhibitory effect on the AKR1C3 was more pronounced. In addition, the molecular docking results suggested that the genistein had a strong affinity with the AKR1C3, and that it could be a promising AKR1C3 inhibitor.

**Conclusion:**

Genistein inhibits the progression of CRPC via the suppression of AKR1C3.

**Figure:**
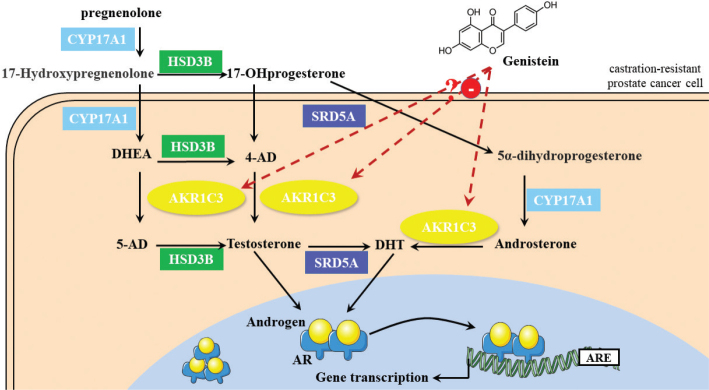

## Popular scientific summary

- Genistein inhibits the progression of castrate resistant prostate cancer (CRPC).
- Genistein inhibits CRPC cell proliferation and in vivo tumorigenesis via suppressing AKR1C3.
- Molecular docking technique reveals the structural basis of AKR1C3 enzyme activity inhibition by genistein flavonoids for the first time.

Prostate cancer is a common tumor among middle-aged and elderly males (1) and is the second leading cause of death in men globally (2). Prostate cancer is a type of androgen-dependent tumor, and its occurrence and progression depend on androgen. Early detection of androgen deprivation can effectively inhibit the growth of prostate cancer, but it has been confirmed that following androgen deprivation therapy (ADT), prostate cancer can develop into castration-resistant prostate cancer (CRPC) (3). With the development of CRPC, the patient’s chance of survival is significantly reduced, and recent research has, thus, largely focused on this disease (4). Patients with this disease now have a number of approved treatment options, including chemotherapy drugs (e.g. cabazitaxel and docetaxel) (5), hormone inhibitors (e.g. enzalutamide and abiraterone) (6), and tumor vaccines (e.g. sipuleucel-T and radionuclide radium) (7, 8). These drugs are typically used alone or in combination to treat the disease and can be effective in prolonging the patient’s survival time. However, the treatment can involve a number of serious side effects. Therefore, the development of natural products of plant origin has become a new direction for CRPC prevention and treatment.

The AKR1C3 gene plays a vital role in the formation and metabolism of androgens, catalyzing the synthesis of specific androgens (testosterone and dihydrotestosterone [DHT]) in tumor cells. It has been demonstrated that a high expression of AKR1C3 is closely associated with the upregulation of stem cell markers during the transformation of prostate cancer into CRPC (9). The inhibition of AKR1C3 presents a potential strategy for the prevention and treatment of CRPC via inhibiting androgen synthesis in tumors.

In recent years, researchers have developed many types of AKR1C3 inhibitor, including 3-sulfamoylbenzoic acid derivatives, aldehyde ketone-degrading enzymes, 3,(R)-2-(6-methoxy Naproxen-2-yl)butyric acid, and various analogues (10). However, the improved CRPC inhibitors continue to involve toxic side effects in clinical application. The identification of a new potential AKR1C3 inhibitor obtained from natural chemicals is thus crucial. Genistein, a highly active natural phytoestrogen found in soybeans, has been proven to have high inhibitory activity in terms of CRPC cell proliferation (11, 12). A previous study (13) revealed that genistein can markedly inhibit the growth of prostate cancer cells and arrest their cell cycle. Moreover, it was also found that feeding soybean diets to immunodeficiency mice reduced the growth of xenograft tumors, promoted apoptosis of prostate cancer cells, and inhibited angiogenesis.

Genistein has also been reported to significantly reduce matrix metalloproteinase type 2 (MMP-2) expression and serum prostate-specific antigen (PSA) concentrations in prostate cancer patients treated with ADT, suggesting that genistein may combat prostate cancer by affecting protease activity and reducing different markers of prostate cancer progression in humans (14). In addition, various studies (15) have demonstrated that genistein can inhibit tumor cell metastasis via blocking the activation of MMP-2 and the phosphorylation of focal adhesion kinase in human prostate cancer cells. Overall, the previous studies suggest that genistein inhibits the development and progression of prostate cancer; however, whether it inhibits the growth of CRPC through regulating AKR1C3 remains unclear.

In this study, the effects and potential mechanism of genistein on CRPC are explored using in vivo and in vitro experiments in view of ascertaining whether genistein could inhibit the progression of CRPC by reducing the expression of AKR1C3. In addition, the interaction between genistein and AKR1C3 is explored using the Discovery studio 4.5 molecular docking software. Overall, the results indicate that genistein can play a role as an ACR1C3 inhibitor and could be employed in therapeutic approaches in combination with traditional remedies to improve CRPC treatment and prevention.

## Materials and methods

### Reagents

Genistein dry powder was purchased from Med-ChemExpress (MCE, USA), a Roswell Park Memorial Institute (RPMI)-1640 medium and Dulbecco’s modified Eagle’s medium (DMEM) were purchased from Biological Industries (Israel), fetal bovine serum (FBS) was purchased from Gibco (USA), and small interfering ribonucleic acid (siRNA) was acquired from GenePharma (Shanghai, China). The inhibitor (ASP-9521) was purchased from MCE (USA), while the streptavidin-biotin complex (SABC) kit was purchased from Solarbio (Beijing, China), and the ki-67 cell proliferation kit was purchased from Gibco (USA). Western and immunoprecipitated cell lysates were purchased from Biyuntian (Beijing, China); the bicinchoninic acid (BCA) protein quantification kit and Tween-20 were purchased from Solarbio (Beijing, China); the PSA/AKR1C3 monoclonal antibody was purchased from Abcam (USA); beta (β)-ACTIN monoclonal, rabbit secondary, and mouse secondary antibodies were purchased from Zhongshan Jinqiao (Beijing, China); a western chemiluminescence horseradish peroxidase substrate was purchased from Millipore (USA); and a 4x loading buffer was purchased from Solarbio (Beijing, China).

### Animals and experimental protocols

Sixteen 4-week-old female BALB/c-nu mice were purchased from Charles River Laboratories and were raised in the specific pathogen free animal room at Chengdu Medical College Research Center. The mice were injected subcutaneously with 5 × 10^6^ 22RV1 cells to establish a xenograft tumor model. After 3 days of acclimatization feeding, all the experimental mice were randomly divided into the experimental group and the control group, with eight mice in each group. During the experiment, each group was administered the control diet, while the experimental group was additionally fed with genistein (100 mg/kg.bw/day) via gavage feeding.

The volume of tumors in the mice was measured every 3 days using vernier callipers, with measurements taken in two vertical directions. The following formula was used to estimate the tumor volume: volume = 0.52 × (length) × 2 (width). After 30 days of breeding, the mice were executed, and xenograft tumors were collected for weighing, immunohistochemistry, and ki-67 staining.

### Immunohistochemistry

The tumor tissues were fixed in 10% paraformaldehyde and embedded in paraffin to produce 4-μm sections. Following deparaffinization and rehydration, the tissue sections were treated with 3% hydrogen peroxide to block endogenous peroxidase activity. Following this, the antigen was repaired via microwave treatment before the tissue sections were treated with 0.2% Triton X-100 and 5% bovine serum albumin (BSA), incubated with a rabbit anti-human AKR1C3 antibody at room temperature (37˚C) and then incubated with a goat anti-rabbit biotinylated antibody. Next, the SABC kit was used according to the instructions, and the tissues were counterstained with hematoxylin before microscopic examinations were performed.

### Cell culture

Human CRPC 22RV1 and VCaP cells were purchased from Shanghai Cell Bank of the Chinese Academy of Sciences. The 22RV1 cells were cultured in phenol red-free RPMI-1640 medium (containing 10% activated charcoal-dextran-treated FBS), while the VCaP cells were cultured in phenol red-free DMEM medium (containing 10% activated charcoal-dextran-treated FBS). All cell cultures were incubated at 37˚C under a 5% carbon dioxide atmosphere.

### Cell counting Kit-8 analysis

The human CRPC 22RV1 and VCaP cells were transformed into a 5 x 10^4^ cells/ml suspension with the culture medium, and 100 µl was inoculated in 96-well plates for 24 h. The next day, different concentrations of genistein working solutions (0, 12.5, 25, 50, and 100 μmol/L) were added for incubation for 24, 48, and 72 h, with five replicate wells set for each concentration. Then, the medium was removed, and 10 μL of 3-(4,5-dimethyl-2-thiazolyl)-2,5-diphenyl-2-H-tetrazolium bromide solution was added into each well. After incubation for 4 h, the medium was replaced with 150 μL of dimethyl sulfoxide. The absorbance was measured using a full wavelength microplate reader at 490 nm. The experiment was replicated three times to calculate the cell proliferation rate.

### The ki-67 analysis

The 22RV1 and VCaP cells were transformed into a cell suspension of 2 x 10^4^ cells/mL, and 2 mL was seeded into a six-well culture plate for 24 h. The next day, different concentrations of genistein working solutions (0, 12.5, 25, 50, and 100 μmol/L) were added for incubation for 48 h. After washing with PBS, 4% paraformaldehyde was added and fixed for 15 min before the suspension was washed again with PBS and permeabilized using 5‰TritonX-100 for 30 min. Then, the BSA was blocked at room temperature for 1 h, and the suspension was incubated with a primary antibody at 4˚C overnight and then incubated with a secondary antibody at room temperature for 1 h. Following nucleofection with 4,6-diamidino-2-phenylindole, the results were observed under an inverted fluorescence microscope. The experiment was repeated three times to calculate the cell proliferation rate.

### Quantitative real-time polymerase chain reaction analysis

The 22RV1 and VCaP cells were treated with different concentrations of genistein (0, 12.5, 25, 50, and 100 μmol/L) for 48 h. Following treatment with RNase-free PBS, the cells were placed in 1.5-ml de-enzyme Eppendorf tubes. The total RNA was extracted from the cells using the TRIzol™ reagent, according to the manufacturer’s instructions. The total RNA was then measured via a ND2000 spectrophotometer for its purity, detected for its integrity using agarose gel electrophoresis, and finally reverse transcribed to make complementary DNA. Following primer4.0 and Oligo6.0 verification, the suspension was synthesized by Shanghai Bio-engineering Co., Ltd. Then, real-time fluorescent quantitative polymerase chain reaction (qRT-PCR) analysis was performed using the BIO-RAD CFX Manager Multiplex Real-time Fluorescent PCR System and BIO-RAD’s SsoAdvanced Universal™ SYBR^®^ Green Supermix. The reliability of the reaction was checked via agarose gel electrophoresis at the end of the PCR, and the data were analyzed after the results were validated.

### Transfection

The 22RV1 and VCap cells were seeded in six-well plates at a density of 2 × 10^5^ cells per well. When the cell density reached 80%, the culture medium was replaced with 1.5 mL of phenol red-free RPMI-1640 and DMEM medium. Following this, 5 μg of siRNA and AKR1C3-homo-77, 447, and 1008 were added in combination with 5 μL of the transfection reagent prior to incubation for 6 h. After observing the negative control (NC) wells under fluorescence microscope, if there was fluorescence and uniform distribution in the cells, each well was washed twice with PBS, and the medium was changed to DMEM prior to incubation for 48 h. Following this, the cells were lysed and proteins were extracted for western blot analysis. The siRNA primer sequences of AKR1C3 are shown in Table 1.

**Table 1:** siRNA primer sequences of AKR1C3

siRNA	Primer sequences(5’*→*3’)
AKR1C3-homo-77	CCAAACACCAGUGUGUAAATT UUUACACACUGGUGUUUGGTT
AKR1C3-homo-447	GGAACUUUCACCAACAGAUTT AUCUGUUGGUGAAAGUUCCTT
AKR1C3-homo-1008	CCACCCUAAUUAUCCAUAUTT AUAUGGAUAAUUAGGGUGGTT

### Western blot assay

The 22RV1 and VCaP cells were treated with different concentrations of genistein (0, 12.5, 25, 50, and 100 μmol/L), homo-AKR1C3 siRNA (NC, fluorescently labeled negative control [FAM NC], positive control [GAPDH], 77, 447, and 1008), and the AKR1C3 inhibitor (ASP-9521) for 48 h. Then, the cells were collected and extracted for total protein. Next, the total protein concentration was detected using the BCA kit. Various protein samples (45 μg) were separated via 10% sodium dodecyl sulphate-polyacrylamide gel electrophoresis, and the proteins were transferred to polyvinylidene difluoride membranes. After 90 min of electrical rotation, the samples were placed in 5% skimmed milk powder and closed at room temperature for 2 h. Subsequently, the membranes were incubated with the primary antibodies overnight at 4˚C. After washing three times with tris-buffered saline–Tween 20, the membranes were incubated with the secondary antibodies for 2 h at room temperature and visualized using enhanced chemiluminescence. The above experiments were repeated three times, while the optical density values of the proteins and the internal reference were analyzed using the ImageJ software.

### Molecular modeling

The ligand-receptor binding template for AKR1C3 was searched and downloaded from the Research Collaboratory for Structural Bioinformatic Protein Data Bank homepage and was then imported into Discovery studio software and modified, including in terms of dehydration, hydrogenation, and de-liganding, in preparation for molecular docking. The chemical structure of genistein was downloaded from the ZINC database, with the AKR1C3 inhibitor (ASP-9521) used as a control. After defining the active site for docking in Discovery studio, LibDock was used for fast molecular docking.

### Statistical analyses

All the data were expressed as mean ± standard deviation, and each experiment was repeated a minimum of three times. One-way analysis of variance was performed using the SPSS 22.0 statistical software, with Dunnett’s *t*-test used for inter-group comparisons. A *P*-value of <0.05 was considered to indicate a statistically significant difference.

## Results

### Inhibition of the growth of xenograft tumors and tumor cell proliferation by genistein

**Fig. 1:**
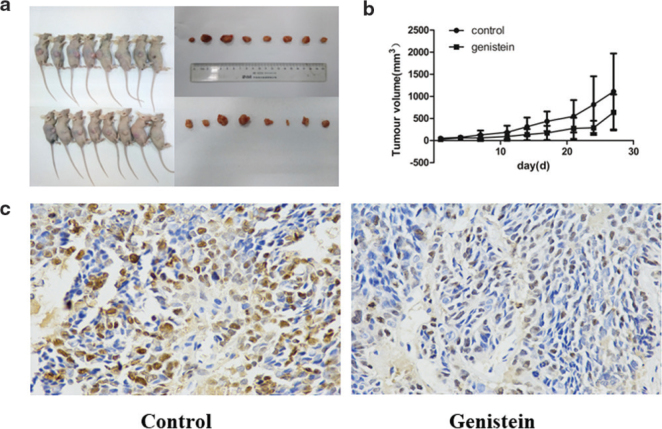
Genistein inhibited the growth of xenograft tumors in mice and tumor cell proliferation. (a) Tumor sizes in mice treated with genistein. (b) Tumor growth curves of mice treated with genistein. (c) Ki-67 determination of positive cell expression in tumor tissues of genistein-treated mice.

To explore the role of genistein in mice xenograft tumors, the mice xenograft tumor model with 22RV1 cells was established, and the experiments were grouped following tumor inoculation growth. The mice in the experimental group were administered 100 mg/kg.bw/day of genistein. As shown in Fig. 1a, the xenograft tumors in the genistein group were smaller and lighter compared to those in the control group. The tumor growth curve (Fig. 1b) also indicated that the genistein inhibited the growth of xenograft tumors in the mice. Furthermore, as shown in Fig. 1c, ki-67 was used to stain two groups of tumor sections separately. The results suggested that ki-67 positive cells were expressed in the tumor tissues of both groups. Compared to the control group, the expression of ki-67 in the tumor tissues was decreased in the genistein group. These results demonstrate that genistein can inhibit xenograft tumor growth and tumor cell proliferation in mice.

### Inhibition of the proliferation and prostate-specific antigen expression of 22RV1 and VCaP cells by genistein

**Fig. 2:**
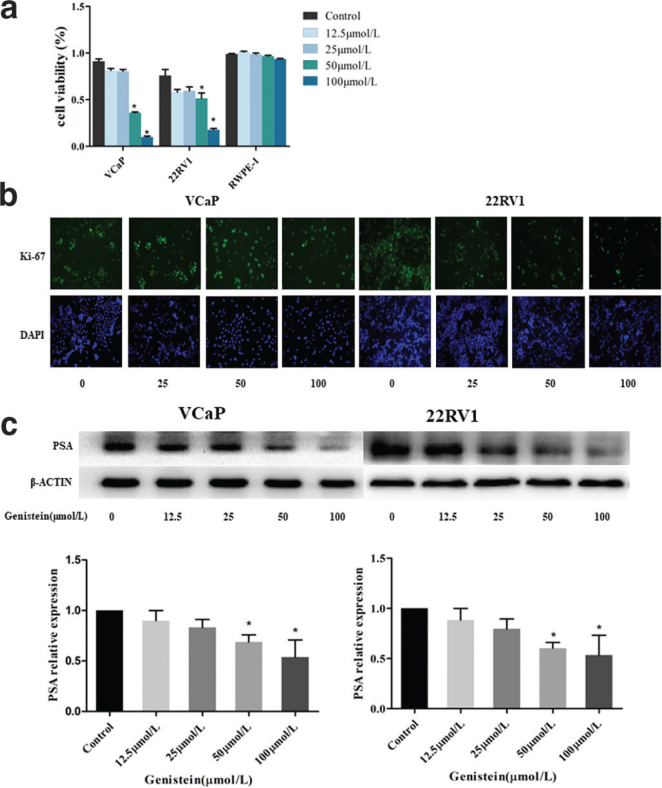
Genistein inhibited the proliferation and PSA expression of VCaP and 22RV1 cells. (a) Proliferative capacity of VCaP and 22RV1 cells after treatment with genistein. * *P* < 0.05 vs. RWPE-1. (b) Ki-67 expression in VCaP and 22RV1 cells after 72h treatment with different concentrations of genistein. (c) Protein expression of PSA in VCaP and 22RV1 cells after genistein treatment. Data represent means ± SD of three independent experiments. * *P* < 0.05 vs. control.

To investigate the effect of genistein on the proliferation of VCaP and 22RV1 cells, two types of CRPC cells were used, with a RWPE-1 cell line used as a control. A Cell Counting Kit-8 (CCK-8) assay and ki-67 analysis were used to detect the proliferation viability of the VCaP and 22RV1 cells following genistein treatment. The CCK-8 assay results indicated that the genistein electively induced VCaP and 22RV1 cell death but exhibited no significant cytotoxicity toward normal human prostate epithelial cells (Fig. 2a). Furthermore, there was an enhancement in the inhibition rate of the value-added viability of these two cell types with the increase in genistein concentration (0, 12.5, 25, 50, and 100 μmol/L), with the inhibition effect found to be significant when the genistein concentration was >50 μmol/L (*P* < 0.05). As shown in Fig. 2b, the Ki-67 assay results also revealed that genistein can inhibit Ki-67 expression in VCaP and 22RV1 cells.

PSA is currently the most widely used tumor marker in the management of early prostate cancer and the attendant follow-up treatment. Elevated PSA levels are associated with an increased risk of developing the disease (16). Therefore, the expression level of PSA at the protein level was also determined via western blot analysis following genistein treatment in both cell types for 48h. The results indicated that genistein could significantly reduce the expression of PSA in a concentration-dependent manner (*P* < 0.05) (Fig. 2 c, d).

### Inhibition of AKR1C3 expression in the mouse xenograft tumors and castration-resistant prostate cancer cell lines by genistein

**Fig. 3:**
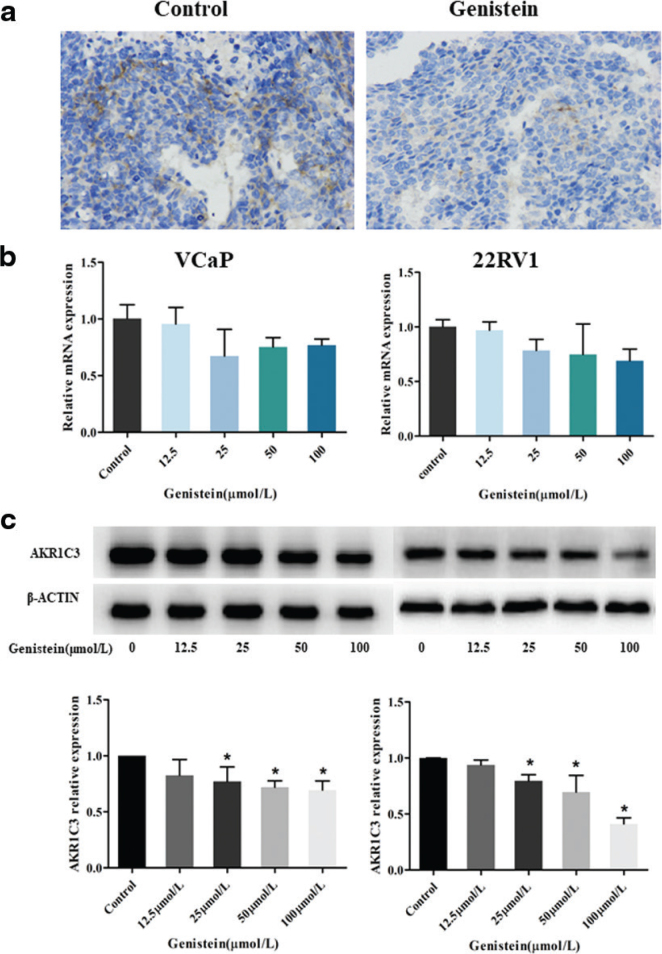
Genistein inhibited the AKR1C3 expression in mouse xenograft tumors and CRPC cell line. (a) Expression of AKR1C3 in tumor tissues of mice after genistein treatment. (b) Quantitative polymerase chain reaction analyses of mRNA expression in control and genistein-treated. Data are presented as the means ± SD. * *P* < 0.05 vs. control. (c) Protein expression of AKR1C3 in VCaP and 22RV1 cells after genistein treatment. Data represent means ± SD of three independent experiments. *P < 0.05 vs. control.

To ascertain what caused the inhibition of both xenograft tumor growth and VCaP and 22RV1 cell proliferation, immunohistochemistry analysis was performed. As shown in Fig. 3a, the expression of AKR1C3 in the xenograft tumor tissues in the genistein group was decreased compared with the control group. Based on this finding, it was hypothesized that the inhibition of xenograft tumor growth was caused by a reduced expression of AKR1C3.

The qRT-PCR and western blot methods were used to detect the expression of AKR1C3 in the 22RV1 and VCaP cells cultured in vitro. First, the level of AKR1C3 expression in normal CRPC cells was determined, and the results indicated that AKR1C3 was highly expressed in both the 22RV1 cells and the VCaP cells (Fig. 3b, c). Following this, the effect of genistein on the expression of AKR1C3 in the 22RV1 and VCaP cells was investigated. The relative expression levels of AKR1C3 in the CRPC cell lines are shown in Fig. 3b. The messenger RNA expression levels of the VCaP and 22RV1 cells following treatment with different concentrations of genistein were lower compared to the control group (*P* > 0.05; but close to 0.05). As shown in Fig. 3c, the protein expression levels of AKR1C3 in the VCap cells treated with genistein concentrations of 12.5, 25, 50, and 100 µmol/L were only 82.6% (*P* > 0.05), 77.0% (*P* < 0.05), 72.0% (*P* < 0.05), and 69.4% (*P* < 0.05), respectively, of those in the control group. Compared with the control group, the protein expression levels of AKR1C3 in the genistein-treated 22RV1 cells were also reduced to varying degrees: 93.9% (*P* > 0.05), 79.6% (*P* < 0.05), 69.6% (*P* < 0.05), and 41.2% (*P* < 0.05). These results demonstrated that the genistein decreased the expression of AKR1C3 in the 22RV1 and VCaP cells, and that the reduction became more obvious as the concentration of genistein was increased.

### Inhibition of AKR1C3 expression in the VCaP and 22RV1 cells by AKR1C3 small interfering ribonucleic acid, the AKR1C3 inhibitor, and genistein, alone or in combination

To further evaluate whether the genistein-induced inhibition of VCaP and 22RV1 cell proliferation is due to the regulation of AKR1C3 expression, the VCaP and 22RV1 cells were treated with AKR1C3 siRNA, the AKR1C3 inhibitor (ASP-9521), and genistein, either alone or in combination. First, the AKR1C3 siRNA with the strongest inhibitory effect on the AKR1C3 expression in VCaP and 22RV1 cells was determined via a western blot assay. The results indicated that AKR1C3 siRNA can reduce the expression level of AKR1C3 in VCaP and 22RV1 cells, and that the AKR1C3 siRNA 447 sequence had the strongest inhibitory effect on AKR1C3 expression (*P* < 0.05), and this was thus adopted for the follow-up experiments.

**Fig. 4:**
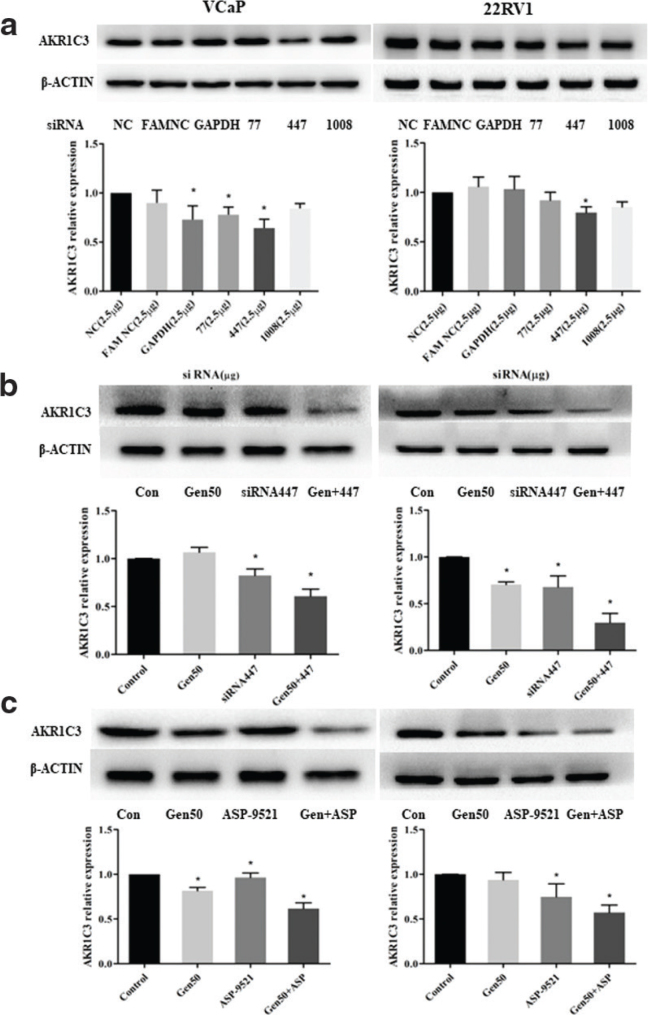
AKR1C3 siRNA, AKR1C3 inhibitor, and genistein alone or in combination inhibited AKR1C3 expression in VCaP and 22RV1 cells, respectively. (a) Expression of AKR1C3 in VCaP and 22RV1 cells after AKR1C3 siRNA treatment. (b) AKR1C3 siRNA 447 and genistein alone or in combination inhibited AKR1C3 expression in VCaP and 22RV1 cells. (c) AKR1C3 inhibitor and genistein alone or in combination inhibited AKR1C3 expression in VCaP and 22RV1 cells. Data are presented as the means ± SD from at least three independent experiments. * *P* < 0.05 vs. control.

Next, the VCaP and 22RV1 cells were treated with 5 μg of AKR1C3 siRNA 447, 50 nmol/L of the AKR1C3 inhibitor (ASP-9521), and 50 μmol/L of genistein, either alone or in combination, for 48 h. Then, western blot analysis was performed to determine the expression of AKR1C3 in the VCaP and 22RV1 cells. As shown in Fig. 4b and Fig. 4c, the expression of AKR1C3 was reduced in both the VCaP cells and the 22RV1 cells in the sample group compared with the control group. Furthermore, when the AKR1C3 siRNA 447 and the AKR1C3 inhibitor were combined with genistein (50 μmol/L), the inhibitory effect on the AKR1C3 was stronger (*P* < 0.05). Thus, it was inferred that the mechanism of AKR1C3 inhibition by genistein is similar to that of AKR1C3 siRNA and the AKR1C3 inhibitor (ASP-9521).

### Target prediction of genistein based on the molecular docking method

**Fig. 5:**
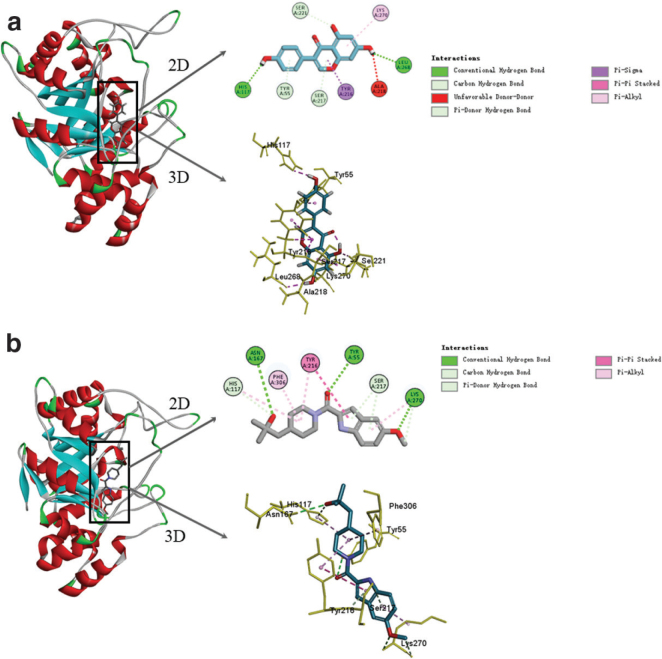
Molecular docking models of genistein with AKR1C3 and AKR1C3 inhibitors with AKR1C3. (a) The three-dimensional and two-dimensional structure of the interaction between genistein and AKR1C3. (b) The three-dimensional and two-dimensional structure of the interaction between AKR1C3 inhibitors and AKR1C3.

To gain further insight into the interaction between genistein and AKR1C3, the attendant interaction mechanism was simulated using molecular docking techniques. Here, the Discovery studio 4.5 software was used for the docking, and the AKR1C3 inhibitor (ASP-9521) was used as a control. The results (Fig. 5) indicated that both the genistein and the AKR1C3 inhibitor could access the active center of the AKR1C3. As shown in Fig. 5a, the genistein interacted with eight amino acid residues of the AKR1C3 (HIS117, LEU268, SER221, TYR55, SER217, TYR216, LYS270, and ALA218) via hydrogen bonding, pi-sigma, pi-alkyl, and pi-pi stacking, while the AKR1C3 inhibitor interacted with the AKR1C3 (Fig. 5b) via hydrogen bonding, pi-pi stacking, and pi-alkyl interaction. The molecular docking scoring function indicated that the -CDOCKER energy value between the genistein and the AKR1C3 was 30.87 kcal/mol, while that between the inhibitor and the AKR1C3 was 22.26 kcal/mol. Under the same conditions, lower values of -CDOCKER energy indicate tighter binding of the receptor to the ligand, suggesting that the genistein was bound more firmly to the AKR1C3 than the inhibitor.

## Discussion

Some reports have suggested that the intra-tumor conversion of adrenaline and the de-novo synthesis pathway of steroids are key factors in the progression of CRPC (17, 18). The AKR1C3 gene, also known as type 5, 17β-hydroxysteroid dehydrogenase (17β-HSD), is a key enzyme in the last two steps of testosterone and DHT synthesis in tumor cells (19, 20), with DHT deemed to promote the growth, proliferation, and metastasis of prostate cancer cells. There are three pathways to synthesize DHT in prostate cells, and AKR1C3 plays an important role in all of them (21). It has been reported that the expression of steroidogenic enzymes such as AKR1C3 becomes upregulated following ADT for prostate cancer patients (22). This indicates that the overexpression of AKR1C3 may be a biological marker for the conversion of prostate cancer from hormone-dependent to CRPC. Therefore, the inhibition of AKR1C3 expression and the attendant androgen synthesis in tumor cells can be used as a therapeutic target against CRPC. This has led to a growing interest among researchers for developing AKR1C3 inhibitors.

Kafka et al. (23) found that MF-15, a compound derived from a plant herb, exhibited strong antitumor properties in various prostate cancer cell models. This compound not only has a strong effect on the androgen receptor (AR) signaling pathway but also reduces the expression of both the AR and AKR1C3, which has the dual effect of inhibiting both androgen biosynthesis and AR signaling cascade in tumors. Elsewhere, Endo et al. (24) discovered a novel and efficient AKR1C3 inhibitor, N-(4-fluorophenyl)-8-hydroxy-2-imino-2h-chromene-3-carboxamide (2d), and subsequently synthesized its derivatives. The authors’ selective detection of AKR1C3 was 220-fold higher than that of other AKR1C isomers. These compounds may be useful for treatment and/or prevention of CRPC. Meanwhile, Zhou et al. (25) designed a low systemic toxicity and a high targeting drug delivery system (CS-4D5/6e) by combining a mansonone derivative and an AKR1C3 inhibitor, 6e, with 4D5-modified chitosan. The system can effectively combat CRPC via downregulating AKR1C3-mediated testosterone levels in tumors. Overall, these studies have demonstrated that the inhibition of AKR1C3-mediated hormone synthesis can suppress the growth of CRPC, and that plant-derived AKR1C3 inhibitors may have similar effects against CRPC.

Genistein exhibits a wide variety of molecular effects, such as inhibiting inflammation, promoting apoptosis, and regulating steroid hormone receptors and metabolic pathways, but it is best known for its ability to inhibit the progression of cancer (26). In fact, various studies have revealed that genistein exerts good prevention and treatment effects in various diseases, including prostate cancer, kidney cancer, nasopharyngeal cancer, colorectal cancer, and breast cancer (27, 28). It has been reported that genistein may inhibit the proliferation of prostate cancer cells through regulating the expression of ER-akt, PSA, p21, Cyclin D1, CDK4, etc. (29). Meanwhile, a double-blind phase II clinical trial confirmed the safety and efficacy of genistein in patients with prostate cancer (30). The results of the clinical studies initially confirmed the potential of genistein as a chemopreventive agent, thus encouraging further research.

In the present study, the growth of xenograft tumors in mice treated with genistein was inhibited, and the expression of AKR1C3 was reduced in various tissue sections of transplanted tumors. These results suggest that genistein may inhibit the proliferation of CRPC by targeting AKR1C3. The in vitro experiments also revealed that 50 μmol/L of genistein can significantly inhibit the growth of 22RV1 and VCaP cells and the expression of PSA in mRNA, as well as the protein levels.

It has been reported that elevated intra-tumor androgen levels and high transcriptional activity of the AR signaling pathway are important factors in promoting prostate cancer progression. Furthermore, homo-AKR1C3 siRNA and specific AKR1C3 inhibitors suppress AKR1C3 expression in CRPC cells through reducing the intracrine androgen levels and AR activity (31). The present in vitro experiments revealed that homo-AKR1C3 siRNA and the AKR1C3 inhibitor, ASP-9521, treated with genistein alone or in combination yielded results that were consistent with those obtained following treatment with either substance alone. Thus, it was inferred that genistein acts in a similar manner to the other two substances, that is, genistein can suppress CRPC proliferation by inhibiting the AKR1C3-mediated T-synthesis pathway. In the molecular docking experiments, the CDOCKER module was used to verify that both genistein and the inhibitor could bind to AKR1C3. In addition, the CDOCKER potentials of various ligands and receptors were assessed, with the results demonstrating that the potential energy of genistein was lower than that of the inhibitor.

The present study demonstrated that the genistein inhibited prostate cancer cell growth via decreasing the expression of AKR1C3 both in vitro and in vivo. The likely attendant mechanism was similar to that of the homo-AKR1C3 siRNA and the ASP-9521 AKR1C3 inhibitor. In summary, edible plant-derived compounds can be used for cancer treatment and provide new ideas for the clinical use of endocrine therapy for the treatment of CRPC.

## Conclusion

The results of this study demonstrated that genistein inhibits the progression of CRPC via decreasing the expression of AKR1C3, providing new evidence for the use of natural flavonoids targeting CRPC.

## Ethics statements

The animal experiments were performed according to the Chinese National Standard ‘Experimental Animals and Environmental Facilities’ and were approved by the Animal Research Committee of Chengdu Medical College.

## Authors’ contributions

YZ designed the study. XY, JY, YL, JC, and TF conducted the research and analyzed the data. XY, JY, TF, and JC wrote the manuscript. YL, LZ, and YZ coordinated and helped to draft the manuscript. All authors read and approved the final manuscript.

## Conflicts of interest and funding

The authors declare that there are no conflicts of interest. The authors have not received any funding or benefits from industry or elsewhere to conduct this study.
